# An Unusual Case of Giant Cell Arteritis

**DOI:** 10.7759/cureus.26483

**Published:** 2022-07-01

**Authors:** Nitasha Goyal, Arjun Basnet, Thai T Donenfeld, Kripa Tiwari, Britney M Clemen, Htin Kyaw, Ifeanyi Nwosu, Emeka C Ibeson, Sarita Konka

**Affiliations:** 1 Internal Medicine, State University of New York (SUNY) Downstate, Brooklyn, USA; 2 Internal Medicine, Maimonides Medical Center, Brooklyn, USA; 3 Internal Medicine, Kathmandu Medical College, Kathmandu, NPL; 4 Internal Medicine, Maimonides Medical Center, New York, USA; 5 Medicine, Maimonides Medical Center, Brooklyn, USA; 6 Rheumatology, Maimonides Medical Center, Brooklyn, USA

**Keywords:** large vessel vasculitis, jaw pain, headaches, rheumatology, giant cell arteritis

## Abstract

Giant cell arteritis (GCA), also known as temporal arteritis (TA), is a systemic autoimmune inflammation of medium and large arteries. It is the most common vasculitis affecting adults older than 50, with an incidence of 20/100,000 and an average age of onset of 70. Typically, patients initially present with new-onset headaches, visual changes and disturbances, jaw claudication, arthralgias, and tender or swollen temporal or occipital arteries. Our patient is a 73-year-old male who presented to the emergency room with 10 days of bilateral headache radiating to the occipital area associated with fevers, persistent chills, generalized weakness, and a headache described as constant, dull, 9 out of 10 pain, and minor pain with neck flexion. Lab work revealed an elevated erythrocyte sedimentation rate (ESR) and C-reactive protein (CRP). The patient had tender palpation to his temples and due to a high suspicion of giant cell arteritis, he was started on high-dose steroids with rapid relief of his symptoms. Biopsy showed evidence of active non-granulomatous vasculitis and confirmed bilateral temporal arteritis within the context of the clinical setting. GCA patients are more likely to be women and typically present with unilateral headache (66% of GCA), jaw claudication (50%), fevers (50%), and transient visual loss (16-54%). Here, we describe a 73-year-old male with a past medical history of cerebral vascular accident (CVA), diabetes, and cancer that presented with 10 days of bilateral headaches and fevers. Unlike the usual presentation, our patient denied any vision and joint pain changes, and the temporal arteries were not stiff to palpation. This patient presentation is unique to previous reports in the limited display of symptoms and absence of the most commonly associated manifestations. Although his presentation supported GCA, the features of elevated ESR and CRP, headache, and fever were too general to diagnose GCA exclusively, and his additional symptoms of rhinorrhea and sinus pain more likely supported infection. Our case indicates the importance of maintaining a high index of clinical suspicion for GCA in the elderly population presenting with headaches and elevated ESR and CRP. GCA, also known as temporal arteritis (TA), is a systemic autoimmune inflammation of medium and large arteries. Typically, patients initially present with new-onset headaches, visual changes and disturbances, jaw claudication, arthralgias, and tender or swollen temporal or occipital arteries. Diagnosis requires high clinical suspicion, and treatment revolves around high doses of steroids.

## Introduction

Giant cell arteritis (GCA), also known as temporal arteritis (TA), is a systemic autoimmune inflammation of the medium and large arteries. It is the most common vasculitis affecting adults older than 50, with an incidence of 20/100,000 and an average age of onset of 70. Women are affected at a higher rate than men, with ratios between 3:1 and 6:1 [1-2]. Clinically, GCA can present gradually or abruptly. Typically, patients initially present with new-onset headaches, visual changes and disturbances, jaw claudication, arthralgias, and tender or swollen temporal or occipital arteries. Other symptoms include low-grade fever, malaise, and scalp pain [1-7]. We describe a patient that presented with only bilateral headache and fevers but ultimately was diagnosed with bilateral GCA.

## Case presentation

The patient was a 73-year-old male who presented to the emergency room with 10 days of bilateral headache radiating to the occipital area associated with fevers, persistent chills, and generalized weakness. On arrival, the patient described the headache as constant, dull, with 9 out of 10 pain, and had minor pain with neck flexion. Lumbar puncture was considered but not performed due to active clopidogrel use. He denied any vision changes, double or stacked vision, loss of vision, floaters, jaw claudication, trismus, hip or shoulder stiffness, arthralgia, dyspnea, and cough. His past medical history was positive for a cerebral vascular accident (CVA) with residual tremor, insulin-dependent diabetes mellitus with diabetic retinopathy and neuropathy, hypertension, and previous treatment for prostate and skin cancer. Head computed tomography (CT) showed no abnormalities except dense microvasculature and central nervous system atherosclerosis. On initial presentation, his labs and vitals showed an elevated white blood count of 17 K/uL, erythrocyte sedimentation rate (ESR) of 108 mm/hr, C-reactive protein (CRP) of 28.9 mg/L, oral temperature of 100.0 F, blood pressure 125/80 mmHg, heart rate of 108 beats/min, and respiratory rate of 16 breaths/min, meeting the criteria for systemic inflammatory response syndrome (SIRS) and potential sepsis. He was admitted with a working diagnosis of meningitis and started on ceftriaxone, ampicillin, vancomycin, and 10 mg of dexamethasone. At this time, due to the elevated ESR and CRP, temporal arteritis was considered a lower differential that needed to be ruled out.

Once admitted to the internal medicine service, he no longer had a constant headache and instead had swelling, sharp pain, and sensitivity to palpation and erythema of the temporal, periorbital, and glabellar regions. There was also sensitivity to an applied pressure on the frontal, ethmoid, and maxillary sinuses, and he endorsed rhinorrhea. On day one post-admission, the associated erythema and swelling resolved after receiving steroids and antibiotics. Only pain to touch in the temporal areas and sinuses remained, and no overt nodularity of the temporal arteries was identified. As his symptoms subsided, the main differential evolved to sinusitis with temporal arteritis lower on the differentials. A CT of his sinuses was ordered to confirm sinusitis, and rheumatology was consulted to rule out temporal arteritis and consider a bilateral temporal biopsy. Rheumatology assessed that there was some evidence to support temporal arteritis and agreed to pursue a temporal artery biopsy and increased prednisone to 60 mg daily. On day two post-admission, the patient denied pain on palpation of the sinuses with significantly decreased pain in the temporal region. The CT scan showed minimal mucosal disease, and the patient underwent a bilateral temporal biopsy. The patient was discharged with prednisone and told to come back for a follow-up a week later when his biopsy results were ready. Although the results showed no giant cells, there was evidence of active non-granulomatous vasculitis and confirmed bilateral temporal arteritis within the context of the clinical setting.

## Discussion

GCA patients are more likely to be women and typically present with unilateral headache (66% of GCA), jaw claudication (50%), fevers (50%), and transient visual loss (16-54%) [3]. Here, we described a 73-year-old male with a past medical history of CVA, diabetes, and cancer that presented with 10 days of bilateral headaches and fevers. Unlike the usual presentation, our patient denied any vision and joint pain changes, and the temporal arteries were not stiff to palpation. Interestingly, GCA has been shown to have a minor association with CVA [3,8-9]. In one review of 166 cases of GCA, 3% of patients had a stroke [8]. In another report looking at 4,086 stroke patients, only 0.15% had been diagnosed with GCA [9]. Although an uncommon symptom, the patient’s history of CVA could be a manifestation of prolonged GCA and should be considered in pertinent history findings.

GCA is classified into four phenotypes: cranial arteritis, polymyalgia rheumatica, nonspecific systemic inflammatory disease, and large vessel vasculitis [7]. For the latter three, these symptoms and presentations usually precede or coincide with TA. Our patient displays cranial arteritis (temporal sensitivity and headaches) characteristics and nonspecific systemic inflammatory disease (fevers). These symptoms are usually the first signs of TA but are often misattributed until more overt symptoms, such as vision changes and joint pain, are present. If left untreated in our patient, he may have developed visual changes shortly after [3].

Most case reports in recent years associate GCA with varying visual changes [3-5] or overlapping conditions [10-11]. The only other case report with similar findings is from 2002, but that patient also had chewing-induced jaw pain [12]. According to the American College of Rheumatology, the patient met four of the five criteria at initial presentation (Table 1). The patient displayed three of seven key features outlined by the British Society for Rheumatology and British Health Professionals in Rheumatology (Table 1) [7]. This patient presentation is unique to the previous reports in the limited display of symptoms and the absence of the most commonly associated manifestations. Although his presentation supported GCA, the features of elevated ESR and CRP, headache, and fever were too general to diagnose GCA exclusively, and his additional symptoms of rhinorrhea and sinus pain more likely supported infection. Our report supports that patients older than 70, with only constitutional symptoms, bilateral headache, and tenderness, should have temporal arteritis as a predominant differential and treated until proven otherwise.

**Table 1:** Criteria for giant cell arteritis The current criteria for giant cell arteritis [7]

American College of Rheumatology	British Society for Rheumatology and British Health Professionals in Rheumatology
Age of onset greater than 50 years	1. Abrupt-onset headache (usually unilateral in the temporal area)
Onset of new headache	2. Scalp tenderness
Temporal artery abnormality (tender or reduced )pulsation	3. Jaw and tongue claudication
Elevated erythrocyte sedimentation rate, defined as 50 mm/h using the Westergren method	4. Visual symptoms (including diplopia)
Abnormal arterial biopsy	5. Constitutional symptoms
	6. Polymyalgia symptoms
	7. Limb claudication

## Conclusions

Giant cell arteritis (GCA), also known as temporal arteritis (TA), is a systemic autoimmune inflammation of the medium and large arteries. Typically, patients initially present with new-onset headaches, visual changes and disturbances, jaw claudication, arthralgias, and tender or swollen temporal or occipital arteries. Diagnosis requires high clinical suspicion, and treatment revolves around high doses of steroids.
